# In the Wake of Invasion: Tracing the Historical Biogeography of the South American Cricetid Radiation (Rodentia, Sigmodontinae)

**DOI:** 10.1371/journal.pone.0100687

**Published:** 2014-06-25

**Authors:** Rafael N. Leite, Sergios-Orestis Kolokotronis, Francisca C. Almeida, Fernanda P. Werneck, Duke S. Rogers, Marcelo Weksler

**Affiliations:** 1 Department of Biology, Brigham Young University, Provo, Utah, United States of America; 2 Coordenação de Biodiversidade, Instituto Nacional de Pesquisas da Amazônia, Manaus, Amazonas, Brazil; 3 Department of Biological Sciences, Fordham University, Bronx, New York, United States of America; 4 Departament de Genètica, Universitat de Barcelona, Barcelona, Spain; 5 Monte L. Bean Life Science Museum, Brigham Young University, Provo, Utah, United States of America; 6 Departamento de Zoologia, Universidade Federal do Estado do Rio de Janeiro, Rio de Janeiro, Rio de Janeiro, Brazil; University of Florence, Italy

## Abstract

The Great American Biotic Interchange (GABI) was greatly influenced by the completion of the Isthmus of Panama and impacted the composition of modern faunal assemblages in the Americas. However, the contribution of preceding events has been comparatively less explored, even though early immigrants in the fossil records are evidence for waif dispersals. The cricetid rodents of the subfamily Sigmodontinae are a classic example of a species-rich South American radiation resulting from an early episode of North American invasion. Here, we provide a temporal and spatial framework to address key aspects of the historical biogeography and diversification of this diverse mammal group by using mitochondrial and nuclear DNA datasets coupled with methods of divergence time estimation, ancestral area reconstruction and comparative phylogenetics. Relaxed-clock time estimates indicate that divergence of the Sigmodontinae began in the middle–late Miocene (ca. 12–9 Ma). Dispersal-vicariance analyses point to the arrival of a single lineage of northern invaders with a widespread ancestral distribution and imply that the initial differentiation between Central and South America gave rise to the most basal groups within the subfamily. These two major clades diversified in the late Miocene followed by the radiation of main tribes until the early Pliocene. Within the Oryzomyalia, tribes diverged initially in eastern South America whereas multiple dispersals into the Andes promoted further diversification of the majority of modern genera. A comparatively uniform background tempo of diversification explains the species richness of sigmodontines across most nodes, except for two akodontine genera with recent increases in diversification rates. The bridging of the Central American seaway and episodes of low sea levels likely facilitated the invasion of South America long before the onset of the post-Isthmian phase of the GABI.

## Introduction

The Great American Biotic Interchange (GABI) is one of the major biogeographic events that shaped modern faunal communities in the Americas. It involved significant dispersal episodes of a number of taxa between North and South America [1], including mammals [2], [3], birds [4], [5], reptiles and amphibians [6], [7], arthropods [8], [9], and freshwater fishes [10]. The reorganization of faunal assemblages resulting from this biotic upheaval is most strikingly observable in the mammalian fossil record [11]. As a consequence of asymmetrical dispersal dynamics and speciation and extinction rates between northern and southern contingents [3], more than half of the present-day mammalian genera in South America were derived from northern immigrants, contrasted with only 10% of North American genera that have southern ancestry [12]. Possible explanations for this asymmetry involve dissimilar taxon pools, surface area, habitat availability and resource use [2], [13]. Although facilitated since the completion of the Isthmus of Panama at around 3.5 Ma [14]–[16], overseas dispersals prior to the main pulses of the GABI (starting at ∼2.7 Ma) also had an impact on the composition of terrestrial mammal communities as evidenced in mammal-bearing units of North and South America [17]. The first of these records correspond to ground sloths that arrived in North America ca. 9 Ma [18] and a procyonid carnivore in South America ca. 7.3 Ma [19], or possibly a gomphothere proboscidean in South America ca. 9.5 Ma [20]. However, these dates set only minimum ages for the initiation of the land mammal interchange [21].

Paleogeographic models that explain patterns of dispersal and diversification in mammals, as well as the influence and underlying causes associated with sea level and climate oscillations, past vegetation dynamics, and tectonics have been the subject of continued research in the last few decades (e.g., [11], [21]–[25]). A number of eustatically-controlled episodes of low sea level since the mid-late Tertiary [26], [27] might have facilitated trans-isthmian land mammal displacements regardless of the presence of an overland corridor. In addition, paleoclimatic changes during the late Cenozoic [28], [29] potentially created varying dispersal opportunities for land mammal taxa associated with different plant communities. Furthermore, the Central American isthmus constituted an uplifting tectonic unit from the late Eocene–early Oligocene [30], which greatly reduced the width and depth of the Central American seaway [31]. Although interoceanic circulation between Pacific and Caribbean waters through the Atrato strait in northwestern Colombia [15] lasted until the final closure of the Isthmus of Panama at ∼3.5 Ma [16], [32], the Miocene collision between the Panama volcanic arc and northwestern South America resulted in extensive land emergence and widespread shallowing of marine waters [33], [34].

Therefore, the paleogeography of southern Central America presented environmental conditions that would have enabled dispersal events even prior to the complete closure of the Isthmus of Panama [21]. Nevertheless, additional data are necessary to refine the evolutionary history of key taxa involved in this important biogeographic scenario (e.g., [35]). Cricetid rodents of the subfamily Sigmodontinae are a classic example of a South American radiation ensuing from a northern invasion. Sigmodontines comprise the second-largest muroid subfamily in the world and are the most diverse group of Neotropical mammals [36]. They possess a range of ecomorphological adaptations to arboreal, terrestrial, fossorial and semi-aquatic life styles; having successfully occupied a variety of habitats such as tropical and subtropical forests, savannas, grasslands and deserts [37].

The historical evolution of this impressive mammalian group has been a topic of debate since the early 1950's. Accordingly, three alternative hypotheses have been advanced to explain the biogeographic patterns and diversification of the Sigmodontinae. First, Simpson [38], [39] proposed on the basis of fossil data that sigmodontines invaded South America relatively recently as part of the post-Isthmian phase of the GABI. Some authors [40]–[42] supported his view due to the absence of undoubted older fossil sigmodontines in the South American records and the many forms presumptively assigned to sigmodontine ancestors that evolved in Central America and southern North America during the late Miocene and early Pliocene. This scenario implies that once sigmodontines crossed the land bridge after an initial *ex situ* differentiation of major lineages, there was an explosive radiation in the previously isolated South American continent.

On the other hand, Hershkovitz [22], [43], Savage [44], and Reig [45]–[49] noted that fossil records of alleged sigmodontine ancestors are fragmentary and poorly represented in Miocene strata of North and Central America. In order to explain the extraordinary diversification of the subfamily in South America, they postulated that the southward invasion of an ancestral sigmodontine happened via waif dispersal well before the main episodes of the GABI, during the early–middle Miocene. Hershkovitz and Reig further reasoned that the earliest fossil remains from Argentina resemble forms of extant genera too advanced to represent the first immigrants. Moreover, Reig [46] contradicted Simpson's hypothesis with the description of *Auliscomys formosus* and *Necromys bonapartei* from the Monte Hermoso Formation in Buenos Aires Province, Argentina, ca. 5.28–4.5 Ma (after [50]), which indicate the presence of sigmodontines in South America prior to the Panamanian overland connection (see also [51]–[53]).

Lastly, Marshall [24] attempted to reconcile the first known appearance of undisputed sigmodontines and stem members from the fossil records of both North and South America. According to him, sigmodontines evolved in North America and northern invaders supposedly adapted to forest environments traveled across the isthmian strait via waif dispersal during a eustatic sea-level drop between 5 and 7 Ma. Marshall envisioned that grazing ecomorphs derived from such ancestral lineages inhabited savanna-like ecosystems restricted to northern South America until ∼3.5 Ma. At that time, an open-dry corridor along the eastern Andean slopes, driven by the Northern Hemisphere glaciation [54], [55], would have connected disjunct savanna habitats and enabled sigmodontines with a grazing ecomorphology to spread into southern South America, thus finally reaching the Argentinean deposits.

The phylogenetic approach offers a robust framework for reconstructing central aspects of the historical biogeography of the GABI [11], [12]. Molecular phylogenies constitute a critical component that can shed light on the timing of divergence and spatial patterns of diversification that otherwise would not be possible due to the fragmentary nature of the mammalian (and other groups) fossil records. Phylogenetic comparative methods have been employed to address key evolutionary questions about the tempo and mode of evolution of several taxa involved in the biotic interchange between North and South America (e.g., [5], [6]). Likewise, recent meta-analyses of molecular dating studies indicate that plant and animal dispersal across the Isthmus of Panama occurred prior to the complete formation of the land bridge [56]. Previous authors have used molecular phylogenetics to investigate the enigmatic evolutionary history of sigmodontine rodents [37], [57]-[59]. These studies pointed to an early sigmodontine diversification. However, conclusions are either based on strict molecular clocks and exclusive mitochondrial datasets [37], [57], have a sparse taxon sampling within the Sigmodontinae [58], or make use of limited fossil calibrations among ingroup taxa [59].

Herein, we expand on the historical biogeography of the subfamily by assessing earlier hypotheses proposed to explain the sigmodontine diversification with respect to the GABI. We use nuclear and mitochondrial DNA sequence datasets to reconstruct the phylogenetic relationships of sigmodontines, and employ methods to estimate divergence times, infer ancestral distributions, as well as evaluate significant shifts in the tempo of diversification. The main goal of our study is to provide a robust temporal and spatial framework within which the scope for the remarkable radiation of sigmodontines in South America can be clearly defined. To that purpose we utilize a comprehensive taxon sampling, a large set of fossil calibrations under relaxed molecular clocks, a Bayesian approach to dispersal-vicariance analysis of ancestral areas, and phylogenetic comparative methods of diversification rates based on extant species richness. We address key questions regarding the evolutionary history of sigmodontine rodents in the context of the GABI. When did this group arrive in South America? Where did diversification initially take place? How many ancestral lineages participated in the invasion? Are there extant sigmodontine lineages that are more diverse or impoverished than expected?

## Materials and Methods

### Ethics statement

The safety procedures for handling animals used in this work are in accordance with the guidelines of the American Society of Mammalogists [60]. Tissue samples were obtained from the following institutions: American Museum of Natural History; Angelo State Natural History Collections; Field Museum of Natural History; Institut des Sciences de l′Evolution de Montpellier; Instituto de Ecología y Evolución de la Universidad Austral de Chile; Instituto Nacional de Pesquisas da Amazônia; Laboratório de Citogenética de Vertebrados, Universidade de São Paulo; Lund University; Martin-Luther-Universität Halle-Wittenberg; Monte L. Bean Life Science Museum, Brigham Young University; Museo de Historia Natural La Salle; Museo de Historia Natural, Universidad Nacional Mayor de San Marcos, Lima; Museo de La Plata; Museo Nacional de Historia Natural del Paraguay; Museo Nacional de Historia Natural, Montevideo; Museo di Anatomia Comparata, Università di Roma ‘La Sapienza’; Museu de Zoologia da Universidade de São Paulo; Museu Nacional do Rio de Janeiro; Royal Ontario Museum; Museum of Southwestern Biology; Museum of Texas Tech University; Museum of Vertebrate Zoology, University of California, Berkeley; National Museum of Natural History; Universidade Federal de Minas Gerais; University of Kansas Natural History Museum; University of Michigan Museum of Zoology; University of Vermont.

### Sampling design and data acquisition

We analyzed a total of 66 extant species from 54 genera of sigmodontines, including representatives of all tribes plus four *incertae sedis* genera (sensu [61]). To assess the placement of the Sigmodontinae relative to other subfamilies within the Cricetidae, we also included members of the Arvicolinae, Cricetinae, Neotominae, and Tylomyinae. In addition, we added representatives of other muroid families as the most distant outgroups, namely the Calomyscidae, Muridae, Nesomyidae, and Spalacidae.

We only used samples for which we had sequence data for both protein-coding mitochondrial cytochrome *b* (*cytb*) and nuclear interphotoreceptor retinoid binding protein (*irbp*) genes. All data were generated as part of previous studies (e.g., [62], [63]) and gathered from GenBank except for the new complete *cytb* sequence of *Rheomys raptor*. Each alignment was complete or mostly complete (≥ 90%) for the majority of sequences, but we did include a few pivotal taxa with partial (≥ 60%) sequences. Our taxon sampling with over 80% of the target genera included in this study is comprehensive but at the same time designed to avoid sampling overrepresentation, which may potentially distort diversification rates [64] and divergence time estimates [65]. Hence, we used a single species from each genus to assemble our dataset, except for those genera (1) representing peculiar distributions within the geographic range of the whole subfamily, such as *Nesoryzomys* from the Galápagos Islands and *Oryzomys* from southern United States; (2) comprised of distinct clades within the tribe Thomasomyini [66]; or (3) with fossils that provided calibration points for internal nodes (namely *Akodon*, *Calomys*, *Oligoryzomys*, *Scapteromys* and *Sigmodon*).

DNA extraction, PCR amplifications (thermal profiles and primer combinations), template purification and cycle-sequencing followed laboratory procedures described in Almeida et al. [67] and Weksler [63] for *cytb* and *irbp*, respectively. GenBank accession and specimen voucher numbers as well as the source of samples are listed in Table S1 in the Supporting Information material available online.

### Phylogenetic inference

Multiple sequence alignments were initially performed using ClustalW [68], and inspected manually to refine coding frame and placement of indels as necessary. Average genetic distances (uncorrected *p*-distance) between major clades were calculated with MEGA 5.1 [69]. We inferred the phylogenetic relationships among sigmodontine rodents and other muroid relatives using a maximum likelihood (ML) framework implemented under the rapid hill-climbing algorithm in RAxML v7.2.6 [70]. We estimated the best-scoring ML tree out of 200 random starting trees obtained with maximum parsimony. Joint branch optimization was performed using two distinct gene partitions under the general time-reversible (GTR) model of nucleotide substitution [71] and among-site rate heterogeneity with four categories of the discrete-gamma distribution (Γ_4_) [72]. *Myospalax aspalax* (Spalacidae) was used as outgroup based on previous muroid molecular systematics studies [58], [73]. Node support was assessed through 1,000 nonparametric bootstrap pseudoreplicates [74] and bipartitions values were drawn onto the best-scoring ML tree.

Bayesian analyses were performed using Markov chain Monte Carlo (MCMC) sampling as implemented in BEAST v1.8.0 [75]. We employed the GTR+Γ_4_ model of nucleotide substitution and unlinked gene partitions. Uniform interval priors were assumed for all parameters except base composition, for which we assumed a Dirichlet prior. We performed four independent runs of 25 million generations each with a 5,000-step thinning. All analyses were checked for convergence in Tracer v1.6 [76] by plotting the log-likelihood values against generation time for each run, and the first five million generations were discarded as burn-in. All posterior parameter estimates had effective sample sizes (ESS) above 200 and the remaining trees were used to calculate posterior probabilities for each node.

### Divergence time estimation

We tested for a clock-like evolution of the molecular dataset using a likelihood ratio test (LRT) in MEGA 5.1 [69]. We rejected the presence of a strict molecular clock (see Results), and thus employed two Bayesian relaxed-clock approaches [77] for the estimation of divergence times in sigmodontines.

The method implemented in the program Multidivtime derives a probabilistic model that describes autocorrelated changes in the evolutionary rate among lineages over time [78]. It allows multiple calibration windows and the use of multilocus data with partitioned models, while providing confidence intervals for rate and time estimates. We used the tree topology obtained from RAxML and estimated branch lengths under the F84 model [79] in the program estbranches distributed with the software package. We ran the Markov chain for one million generations sampling at every 100^th^ cycle and discarded a burn-in of 10^5^ cycles. We set the expected number of time units between tip and root to 2.0, and its standard deviation to 0.5. These values refer to 20 ± 5 Ma which is in between the age of the first putative forms assigned to modern cricetids from the Oligocene–Miocene boundary [80]-[82]. Moreover, this date is in accordance with previous age estimates for the basal radiation of the Eumuroida [58], [83]. The mean and standard deviation of the prior distribution for the rate at the root node followed recommendations in the program manual and were given by averaging the median distances between the root and tips, calculated for each gene with the R package ‘adephylo’ [84], divided by the time unit where one unit equals 10 million years.

We also estimated divergence times using a relaxed molecular clock framework that allows evolutionary rates to vary along the tree branches under an uncorrelated lognormal relaxed-clock model [85], as implemented in BEAST (see Phylogenetic inference section above for parameter settings). We used lognormal prior distributions to constrain the nodes with fossil calibrations [86].

**Figure 1:**
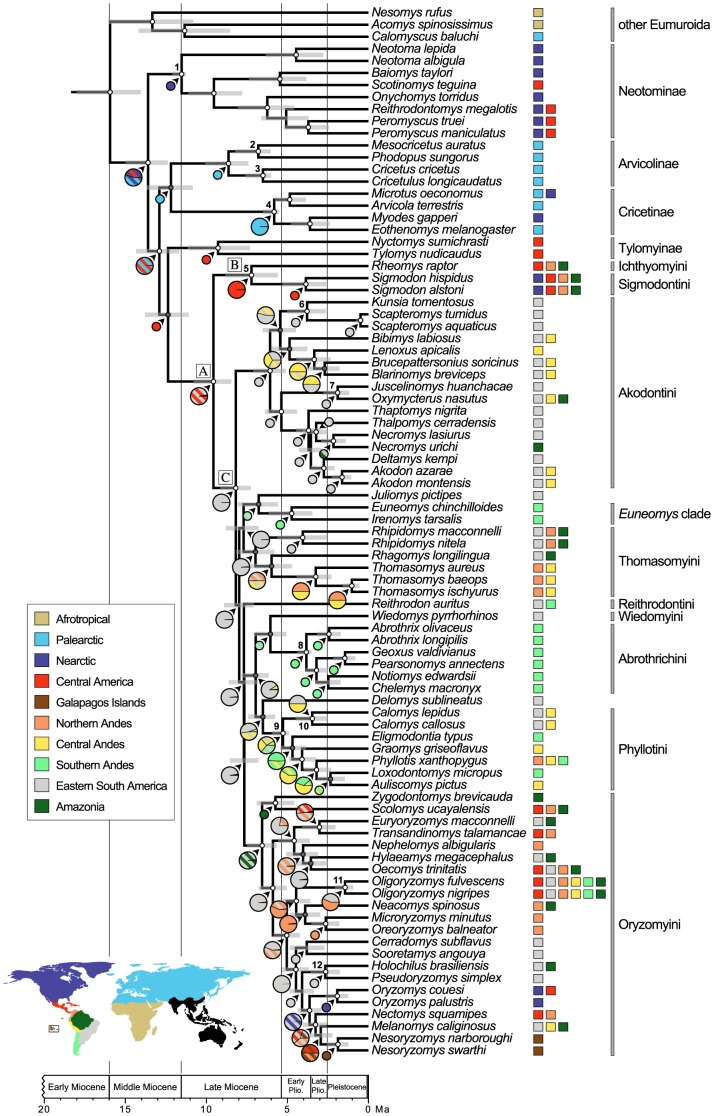
Time-calibrated phylogeny and ancestral range reconstruction of the subfamily Sigmodontinae. Divergence ages were estimated using BEAST relaxed-clock model; node bars are 95% highest probability density credibility intervals. Pie charts represent the marginal probability of ancestral ranges reconstructed for each node of the phylogeny using S-DIVA; stripped diagonals indicate ancestral ranges of two areas and nodes with maximum probability reconstructions are downsized for clarity. Posterior clade probabilities are depicted with circles at the nodes with PP ≥ 0.95 (white) and PP ≥ 75% (gray). Node numbers correspond to fossil calibrations listed in Table S2. (A) Sigmodontinae crown; (B) Sigmodontalia; (C) Oryzomyalia.

We incorporated information from a total of 12 fossils to calibrate the nodes of the phylogeny (Figure 1 and Table S2). These nodes represented multiple shallow (younger) and deep (older) calibrations, including crown clades, to avoid potential pitfalls of divergence time estimation associated with the number and distribution of time constraints across nodes [87]. In addition, we calibrated only well-supported nodes for those fossils that we had strong paleontological evidence of their clade membership and taxonomic status. Moreover, we assessed the consistency of fossil calibrations using a jackknife approach in which Multidivtime pseudoreplicates were performed removing each calibration point at a time.

### Dispersal-vicariance analysis

We investigated the biogeographic history of sigmodontine rodents with S-DIVA [88] in RASP [89], which is a Bayesian approach to dispersal-vicariance analysis (DIVA). In DIVA, ancestral areas are reconstructed onto the nodes of a fixed topology under a maximum parsimony criterion that minimizes the number of dispersal and extinction events and that assumes speciation results from vicariance of widespread species [90]. In S-DIVA, uncertainty is taken into account with a set of phylogenetic trees and alternative ancestral ranges at each node are averaged over all trees weighted by the frequency of that node in ancestral reconstructions. We used a total of 9,000 tree topologies from the posterior distribution of trees inferred in BEAST and *maxarea*  =  2 or 3. The range of the areas assigned to each terminal taxa corresponds to their geographic ranges at the generic level, which encompasses overall common distribution limits for the genera among ten major areas, namely: Afrotropical, Palearctic, Nearctic, Central America, Eastern South America, Northern Andes, Central Andes, Southern Andes, Amazonia, and Galapagos Islands (see map in Figure 1).

### Phylogenetic comparative analyses

Phylogenetic comparative analyses were based on the chronogram tree comprised of the 66 sigmodontine terminals extracted from the dated phylogeny obtained with BEAST (i.e., after other muroid taxa were pruned away). We explored the tempo of increase or decrease in species richness as a function of diversification within the subfamily by plotting the number of lineages observed through time on the phylogeny (lineages-through-time plot: LTT). We also used MEDUSA comparative algorithm [91] to investigate if the extant diversity of the Sigmodontinae is explained by background rates of speciation and extinction, or if significant shifts in diversification rates occurred along generic and tribal lineages. The method accommodates branch lengths and incomplete sampling using taxonomic richness information to detect nodes leading to large clades also with short waiting times. MEDUSA integrates taxonomic richness data in a stepwise approach fitting probabilistic models for the backbone phylogenetic tree with subsequently complex models, and then uses the corrected Akaike information criterion (AICc) [92] to contrast and choose the best-fitting rate shift model. We fitted birth-and-death as well as pure-birth (Yule) models, and the AICc threshold was calculated automatically with the threshold selection function. The richness dataset was generated using the number of species per each genus, as compiled from Musser and Carleton [36] and amended with new species descriptions from the literature. In addition, the 66-taxa tree was pruned down to 54 terminals representing unique sigmodontine genera. We performed comparative diversification analyses in R 3.0.2 [93] with packages ‘geiger’ [94], ‘ape’ [95], ‘picante’ [96], ‘phytools’ [97] and ‘laser’ [98].

## Results

### Sequence data

The complete dataset comprised a total of 88 taxa including nearly 75% of extant sigmodontine genera and all tribes plus representatives of other cricetid subfamilies and major muroid clades. Aligned nucleotide sequences were 1,140 and 1,236 bases long for the mitochondrial *cytb* and nuclear *irbp* genes, respectively. The concatenated sequence matrix contained a total of 1,262 variable sites and 1,002 parsimony informative sites. The *cytb* partition contributed 646 variable and 572 parsimony informative characters, whereas *irbp* contributed 616 variable and 430 parsimony informative characters. Average pairwise sequence divergence (uncorrected *p* distance) based on *cytb* data ranged from 17 to 26% between tribes or *incertae sedis* genera; for *irbp*, average pairwise sequence distance between tribes or *incertae sedis* genera ranged from 3 to 7%. Mean sequence distance between the Oryzomyalia and its sister clade was 20% and 6%, for *cytb* and *irbp*, respectively. Distances between the Sigmodontinae and other cricetid subfamilies varied from 20 to 24% for *cytb*, and from 7 to 9% for *irbp*.

### Phylogenetic relationships

The Sigmodontinae was recovered as a well-supported monophyletic clade in the maximum likelihood and Bayesian phylogenetic analyses with a bootstrap value (BS) of 100% and posterior probability (PP) equal to 1.00, respectively (Figures S1 and S2). Two major clades within the subfamily also were recovered with high nodal support in both phylogenetic inferences (BS  =  100%; PP = 1.00): (1) the Sigmodontalia, new taxon herein defined as the most recent common ancestor of the tribes Sigmodontini and Ichthyomyini and all of its descendants, primarily distributed in Central America and southern North America; and (2) the Oryzomyalia (sensu [58]), which is the most diverse and widespread clade including all sigmodontines except for the Sigmodontalia and has a distribution chiefly in South America.

Basal relationships within the Oryzomyalia are poorly resolved, in agreement with previous molecular phylogenetic analyses that produced similar results under different sampling designs [37], [58], [59], [62], [63], [73], [99]. The tribes Abrothrichini, Akodontini, Oryzomyini and Phyllotini were consistently recovered as well-supported monophyletic clades (BS ≥ 95%; PP  =  1.00), but the monophyly of Thomasomyini (sensu [100]) was recovered only in the Bayesian inference and with little nodal support (PP  =  0.81) (Figure S1)

Six genera including *Reithrodon*, *Rhagomys* and four others regarded as *incertae sedis* (Table S1) have ambiguous phylogenetic placements and negligible support values. However, *Euneomys* and *Irenomys* were recovered as sister taxa in both inferences with high nodal support (BS  =  99%; PP  =  1.00; Figure S1). This clade is hereafter termed the *Euneomys* clade.

The subfamilies Sigmodontinae and Tylomyinae were recovered as sister clades with moderate levels of support (BS  =  74%; PP  =  0.91; Figure S1). Basal relationships between the Sigmodontinae and other cricetid subfamilies, the Arvicolinae, Cricetinae and Neotominae, overall are poorly resolved; a result that is in agreement with those of other studies using additional nuclear genes [58], [83], [101].

### Divergence time estimates

The hypothesis of a strict molecular clock evolution of the phylogeny of sigmodontines plus other muroids was statistically rejected (LR  =  2(29664–29525); *P*≤0.0001). Hence, we estimated divergence times under the assumption of a relaxed molecular clock that incorporates variation in substitution rates across the branches of the phylogeny. Distribution of the rank correlation for rate change between the pair of genes and the *P*-value approximated in the Multidivtime program could not reject the null hypothesis that *cytb* and *irbp* change rates independently (*r* = −0.22; *P* = 0.85).

In general, divergence dates estimated in Multidivtime are older and credibility intervals are broader than those estimated in BEAST (Table 1 and Figure S2). The large credibility intervals estimated under the Multidivtime method could be minimized by using fossil calibrations as maximum age constraints [87], but due to uncertainty of taxonomic affinity we opted out of upper bounds on node ages. These time differences tend to increase at deeper nodes mainly towards the lower limit of credibility intervals. For instance, there is a 4.0-Ma difference between BEAST and Multidivtime estimates for the age of the Cricetidae. However, the time differences are 2.5 Ma for the family Sigmodontinae and 2.0 Ma or less for nodes within the Oryzomyalia. In addition, probability intervals show credibility overlap above 70% for the majority of sigmodontine nodes, except for the Akodontini and Sigmodontini with 62% and 37% overlap, respectively. We attribute these discrepancies to topological differences between the maximum likelihood phylogeny from RAxML used as input in Multidivtime analysis versus the Bayesian phylogeny inferred jointly in BEAST, and the fact that the former method assumes rate autocorrelation whereas the latter employs uncorrelated rates.

**Table 1:** Divergence dates for major sigmodontine clades and the family Cricetidae.

	BEAST		Multidivtime	
Taxon	Time	95% HPD	Time	95% CI
Cricetidae	13.7	12.5–15.1	17.7	13.8–25.0
Sigmodontinae stem	12.4	11.2–13.9	15.5	11.8–22.1
Sigmodontinae	9.6	8.5–10.8	12.1	9.1–17.5
Oryzomyalia	8.2	7.3–9.2	10.2	7.6–14.8
Sigmodontalia	7.2	5.6–8.9	10.0	7.1–14.7
Oryzomyini	6.6	5.7–7.5	7.6	5.5–11.2
Akodontini	6.1	5.1–7.2	8.1	5.9–11.8
Wiedomyini + Abrothrichini	6.6	5.1–7.1	7.6	5.5–11.2
Phyllotini	5.3	4.9–6.0	7.0	5.1–10.2
*Euneomys* clade	4.8	3.5–6.2	6.3	4.3–9.4
Abrothrichini	3.9	3.5–4.4	5.2	3.7–7.8
Sigmodontini	3.9	2.6–5.3	6.3	4.3–9.5

Node ages (Ma) and 95% highest probability density (HPD) and credibility (CI) intervals estimated under rate variation and autocorrelation relaxed molecular clocks with BEAST and Multidivtime programs, respectively.

Dates of divergence estimated in jackknife analyses indicate that fossil calibrations were overall consistent across the nodes of the phylogeny (Figure S2). Divergence of the crown Cricetidae dated to the early–middle Miocene, between 17.7 and 13.7 Ma (as estimated using Multidivtime and BEAST, respectively). The basal radiation among cricetid subfamilies occurred within a relatively short period of time before the end of the middle Miocene. The ancestral sigmodontine lineage split from the tylomyine clade of Middle American endemics between 15.5 and 12.4 Ma, whereas the crown Sigmodontinae diverged from 12.1 to 9.6 Ma. Divergence of Oryzomyalia and Sigmodontalia took place between 10.2 and 8.2 Ma and between 10.0 and 7.2 Ma, respectively, and radiations of major tribes occurred within the late Miocene to early Pliocene (Table 1). Considering observed credibility intervals, the tribes Oryzomyini and Akodontini, which together comprise the greatest diversity of the Sigmodontinae (i.e., more than 50% of genera [61]), started to diversify for the most past before the early Pliocene, whereas divergence of the tribes Phyllotini and Abrothrichini and the *Euneomys* clade mainly preceded the late Pliocene. Initial dates for the tribe Sigmodontini fall within the late Miocene–early Pliocene period, although time estimates may well extend into the late Pliocene.

In sum, most, if not all tribes and genera considered as *incertae sedis*, as well as the majority of sigmodontine lineages at the generic level diverged before the formation of the Panamanian land bridge. Only some splits between genera and intrageneric divergences occurred when an overland connection between the Americas was already in place (i.e., late Pliocene and earlier). Yet, with a few exceptions, credibility intervals for the majority of these nodes encompass older times (Figure 1).

### Ancestral area distributions

The S-DIVA analysis using *maxarea*  =  2 (with *maxarea*  =  3 results are very similar; not shown) suggests that crown nodes of the majority of cricetid subfamilies have a single area of ancestral distribution in either the Nearctic (Neotominae), Palearctic (Arvicolinae and Cricetinae), or Central America (Tylomyinae). The ancestral area of the Sigmodontinae is an exception. Whereas the node connecting both tylomyines and sigmodontines has essentially a Central American ancestral distribution (Figure 1), the most recent common ancestor of the Sigmodontinae underwent an additional dispersal into Eastern South America (ESA). Vicariance then separated this widespread sigmodontine lineage into two major clades, the Sigmodontalia and Oryzomyalia. Within the Sigmodontalia, modern genera presently reach the northern part of South America, but its constituent tribes Sigmodontini and Ichthyomyini are ancestrally distributed in Central America (Figure 1). Within the Oryzomyalia, stem groups have an Eastern South American ancestral distribution whereas tribal lineages spread throughout South America in multiple dispersal episodes. Additionally, the ancestral ranges of the tribes with well-supported nodes are inferred as one single geographic area, except for the Oryzomyini and the Phyllotini.

The most recent common ancestor of the tribe Oryzomyini extended its distribution into Amazonia via dispersal, whereas vicariance subsequently split the ancestor of both *Scolomys* and *Zygodontomys* in Amazonia from the majority of oryzomyines remaining in ESA. Most oryzomyine lineages have ancestral distributions in part or entirely in ESA, although there is some ambiguity among areas in the Northern Andes, Central America and the Galapagos Islands (e.g., *Euryoryzomys* + *Transandinomys*, *Nectomys* + *Melanomys*–*Nesoryzomys*). Also, ancestral and/or current ranges of a number of oryzomyine genera suggest multiple reinvasions of the North American continent (i.e., with distribution in the Nearctic and Central America) dating from the early–late Pliocene boundary onwards, not to mention the arrival of *Nesoryzomys* in the Galapagos during the same period. Therefore, such dispersal episodes occurred relatively recently, by the time when the Panamanian land bridge was already in place.

The Phyllotini have ancestral distributions with ambiguous area combinations of ESA and the Central and Southern Andes. Diversification of the Akodontini began in ESA and gave rise to two major lineages, one with ancestral distributions almost exclusively in ESA (except for *Necromys* additionally in Amazonia) and the other with ambiguous ancestral reconstructions in ESA and the Central Andes. The node connecting the genus *Wiedomys* with the tribe Abrothrichini extended its range into both ESA and the Southern Andes, but the ancestors of the latter have exclusive Southern Andean distributions due to vicariance. Likewise, the ancestral distribution of both *Juliomys* and the *Euneomys* clade encompasses ESA and the Southern Andes while vicariance accounted for the split between *Euneomys* and *Irenomys* in the Southern Andes.

### Phylogenetic comparative analyses

**Figure 2:**
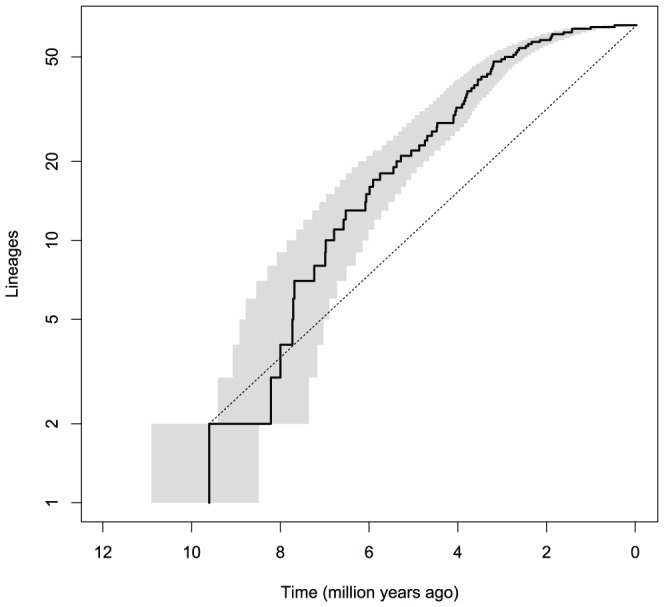
Lineage through time plot of the Sigmodontinae. The number of sigmodontine lineages on a logarithm scale through time inferred from the maximum clade credibility tree (black line) and the confidence interval in terms of time calculated from a collection of maximum probability trees (gray shading) generated in BEAST; expected accumulation of lineages under a constant rate of diversification (dashed line).

The LTT plot is expected to form a straight line when the numbers of lineages are plotted on a logarithmic scale and diversification rates are constant through time [102]. The observed LTT plot lay above this line as indicative of an initial burst of diversification in the sigmodontine radiation. The confidence interval of the LTT plot calculated given the number of lineages in terms of time indicates that such pattern correlates with a period of between-tribe differentiation (Figure 2).

**Figure 3:**
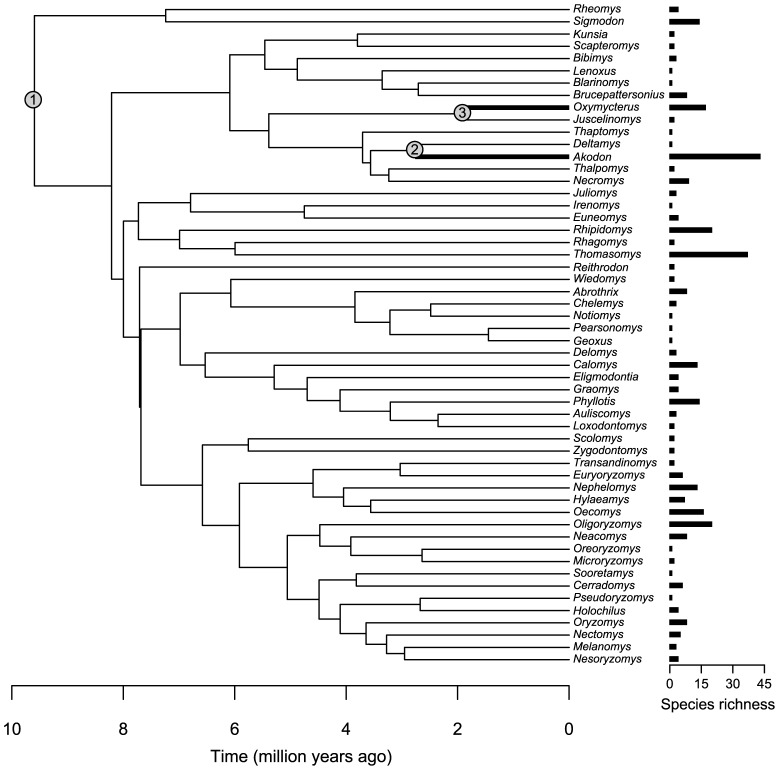
MEDUSA analysis of diversification rate shifts across the generic-level phylogeny of the Sigmodontinae. Node numbers indicate the background tempo of diversification for most sigmodontine rodents (clade 1), and the order at which unusual rate shifts were inferred by fitting MEDUSA stepwise model. Clades 2 and 3 are exceptionally diverse akodontine genera (*Akodon* and *Oxymycterus*, respectively) depicted with thicker lines. Horizontal bars indicate species richness per each genus.

The optimal MEDUSA model identified two significant breakpoints in diversification rates of the Sigmodontinae (Figure 3), specified by a 5-parameter (Yule) model and AIC threshold equal to 3.1325 (*ln*L  = −204.7474, AICc  =  420.0888). The background tempo of diversification for the majority of nodes in the phylogeny is comparatively modest (*r*  =  0.4727 lineages per million year), in contrast to the nodes leading to the akodontine genera *Akodon* and *Oxymycterus* with increased rates of diversification. These threefold rate shifts occurred independently twice during the late Pleistocene, the first in *Akodon* (*r*  =  1.3584 lineages per million year) and the second in *Oxymycterus* (*r*  =  1.4810 lineages per million year), two of the most diverse sigmodontine genera with 43 and 17 extant species, respectively. In contrast, we did not detect significant shifts in the diversification rates of other species-rich genera, such as *Thomasomys*, *Rhipidomys* and *Oligoryzomys*.

## Discussion

The role of the GABI in shaping the historical biogeography and diversification of sigmodontine rodents has been debated on the basis of extensive paleontological work during the last few decades and, more recently, in the light of molecular phylogenetics. The oldest South American fossil remains found in Argentina and that can be undoubtedly ascribed to sigmodontines date from the late Miocene to early Pliocene [51]–[53], whereas previous phylogenetic studies using different molecular clock methods and sampling strategies (e.g., choice of taxa, molecular markers and fossil calibrations) place the origin of the sigmodontine radiation in the middle to late Miocene [37], [57]–[59], [103]. Here, we endorse both molecular and paleontological studies in that the diversification of sigmodontine rodents predates the main episodes of the GABI initiated in the late Pliocene. This study is based on a comprehensive set of fossil calibrations that produced somewhat younger ages and narrower credibility intervals than previous divergence dates also estimated under an uncorrelated relaxed-clock approach (see [59], [83], [103]). According to our time estimates sigmodontines began to diversify approximately 12 to 9 million years ago. Nevertheless, given the fragmentary nature of the fossil record, it was unclear on the basis of divergence dates alone whether the initial diversification of the subfamily occurred in the North or South America.

In order to clarify this biogeographic conundrum it was therefore necessary to explicitly incorporate spatial information from ancestral distributions. The South American origin of the Oryzomyalia clade has been recently recovered by means of a wide-ranging area assignment used to reconstruct ancestral ranges onto the nodes of the sigmodontine phylogeny [59]. By dividing South America into five distinct areas used in the reconstruction of ancestral ranges (Figure 1), we were able to reveal areas of differentiation and possible routes of dispersal among tribes. Accordingly, stem sigmodontines split from the lineage leading to tylomyine rodents during the transition from middle to late Miocene in Central America, and then they spread into both Central and South America. The subsequent basal split between the Sigmodontalia and the Oryzomyalia, with ancestral distributions confined to Central America and Eastern South America (ESA), respectively, may be linked to an increased uplift of the Eastern Cordillera in the late middle Miocene [104], [105]. In any case, a more comprehensive taxon sampling of the Sigmodontalia, particularly of genera in the tribe Ichthyomyini, would be necessary to attest whether additional ancestral sigmodontine lineages entered South America as early as the late Miocene or only later. Regarding further reinvasions of the North American continent by oryzomyines, it is likely that such south-to-north episodes involved overland crossing rather than waif dispersal in the opposite direction given that southern Central America already presented widespread shallow waters by the earliest Pliocene [33] and divergence dates point to a time when the Panamanian land bridge was complete [14].

Our time estimates for the first sigmodontine invasion of the South American continent provide further evidence to warrant a temporal framework that refutes Simpson's late arrival hypothesis [38], [39]. They also contradict Marshal's hypothesis that stem sigmodontines invaded South America favored by eustatically-driven waif dispersal *ca.* 5–7 Ma [24]. Although not as old as envisioned by some early authors (e.g., [22], [44], [48], [49]), divergence dates are in agreement with an early arrival of sigmodontine rodents in South America during the middle to late Miocene (i.e., instead of early–middle Miocene). Alternatively, a regional unconformity in southern Central America dated to 14.8–12.8 Ma separates underlying open marine sequences from depositional units with shallowing upward depths [33]. Moreover, the subfamily divergence followed shortly after a major eustatic lowering of 50±5 m (derived from offshore backstripping and δ^18^O data) [106], [107]. Nevertheless, this first invasion must have happened via waif dispersal rather than by an overland route, since the deepest part of the isthmus still had bathyal depths (> 500 m) in the middle to late Miocene [33].

Sigmodontine ancestors may have crossed the Central American seaway, a ∼200-km wide strait east of the Panama Canal Basin connecting the Pacific Ocean and the Caribbean Sea [30], or alternatively, they may have reached South America by island-hopping through the Antilles [108]. We argue in favor of the former as the most feasible route given that southern Central America formed a subaerial peninsula connected to North America as early as ∼19 Ma [23], [25], [32], whereas the Aves Ridge (SE Caribbean Sea) have undergone increased subsidence and subdivision by the middle Miocene [109], [110]. Also in accordance with this fact is that no significant diversity of sigmodontine rodents or basal cricetid forms exist in the Caribbean. We hypothesize that sigmodontines arrived in northwestern Colombia; most likely atop a raft made of entwined plant material washed ashore in the wake of river floods in the isthmian region. Similarly, transoceanic dispersals of caviomorph rodents and platyrrhine primates from Africa have been suggested as mechanisms for the colonization of South America [111], [112]. Besides, there are quite a few examples of sigmodontine taxa inhabiting oceanic islands that support the possibility of waif dispersal (e.g., [113]–[115]).

It thus seems that sea level falls have been instrumental in facilitating the invasion of South America by sigmodontines. Despite the fact that the tectonically active setting of the isthmian region obscures potential relationships between global sea-level changes and the configuration of paleoenvironments in southern Central America [116], low stands of sea level may also have exposed coastal areas with shrub woodland and savanna-like formations [21]. Although late Tertiary palynofloras typically resemble modern tropical communities (e.g., [117]–[119]), volcanic peaks 1400–4000-m high produced a rain-shadow zone within which different paleosols indeed supported a variety of scrublands that are difficult to reconstruct from fossil floral assemblages alone [120]. In such paleogeographic setting characterized by a complex vegetation mosaic and considerable topographic amplitude, increasing elevations would have given rise to more temperate short-lived open habitats [29], [121] under a climate that, albeit a less marked dry season, was overall drier and cooler than today [120]. Hence, pre-Isthmian paleophysiographic conditions promoted habitat diversity and likely played a role on movement patterns of both forest- and savanna-adapted mammals [122].

Members of the subfamily Tylomyinae, which are consistently recovered as the sister-group of the Sigmodontinae [58], [83], are inhabitants of mesic forests in Central America, whereas other cricetid subfamilies (Neotominae, Cricetinae and Arvicolinae) essentially occur in temperate ecosystems of North America and the Old World more similar to tropical and subtropical open country. Accordingly, the stem lineage of the Sigmodontinae may possibly have evolved into a forest ecomorph resembling modern tylomyines, or alternatively may have retained an ecomorphology characteristic of temperate environments common to cricetids elsewhere. In the latter case, a likely dispersal route would encompass relict shrub and savanna woodlands along the Caribbean coast and Llanos region of Colombia and Venezuela and the Guiana plateau, which at times during the Tertiary would have been connected to open-dry formations of central and northeastern Brazil [123]–[126]. As a matter of fact, savanna woodland vertebrates are represented in diverse fossil assemblages from the Miocene records of Colombia and Venezuela [127], [128]. It is also noteworthy that the timing of divergence for the subfamily agrees with an ice-growth phase after the middle Miocene Climatic Optimum (17 to 15 Ma) corresponding to cooler and drier climatic conditions, which culminated in the reestablishment of East Antarctic ice sheets by 10 Ma [55] and may have promoted feasible pathways through open-country habitats of South America. Further investigation of ecological and phenotypic traits using methods of ancestral character state reconstruction is needed to ascertain if the sigmodontine ancestor possessed adaptations to sylvatic or pastoral lifestyles.

Based on patterns of diversity and distribution of the Sigmodontinae, Reig [49] proposed that episodic dispersals along a north-to-south axis gave rise to major tribal clades in the Andean mountains, which were regarded as the main areas of differentiation of generic lineages. In fact, several nodes within the subfamily support the view that the Andes provided niche opportunities that ultimately fueled the radiation of a number of sigmodontine genera in the region. Nevertheless, our biogeographic analysis recovered Eastern South America as the chief ancestral area for crown nodes within the Oryzomyalia, pointing that ESA rather than the Andes served as the center of differentiation between tribes in this clade. Subsequent within-tribe differentiation departing from ESA happened in the Andean mountains following multiple independent colonizations by different tribal lineages. This diversification pattern is rather different from that observed in octodontoid rodents, a South American endemic clade with its origin in the southern Andes from where diversifying lineages extended their ranges into other parts of the Andes as well as xeric and mesic habitats of South America [129].

The extant taxonomic diversity of sigmodontine rodents is explained by a comparatively uniform background rate of diversification across most lineages within the subfamily, except for the genera *Akodon* and *Oxymycterus* that are particularly diverse in the Andes and the Atlantic region. Both these widespread akodontine clades exhibited a significant increase in diversification rates and more recently than other sigmodontine genera examined with similar taxonomic diversity. It is important to note that in this study the comparison of among-lineage rate variation is inferred relative to nodes within the crown Sigmodontinae only. Nevertheless, inspection of the LLT plot suggests an initial burst of diversification of tribes once sigmodontines colonized South America, which may explains the low resolution of phylogenetic relationships at the tribal level.

Recent investigations point to rapid rate shifts of the Sigmodontinae when compared to other rodent groups across the Muroidea. Fabre et al. [101] identified ten increased shifts in the subfamily with a topological-based measure of clade imbalance for detecting exceptionally diverse lineages. Schenk et al. [83] indicated a region of increased rate of diversification near the base of the Oryzomyalia which is correlated with the colonization of South America, although the three different comparative methods they employed to a nuclear DNA phylogeny of muroids overall shared no rate transitions among nodes within the Sigmodontinae. Additionally, differences between rate shifts found using the MEDUSA approach here and in Schenk et al. [83], for instance, may be attributed to the use of different genes and taxa with larger representation of the deeper nodes of the muroid phylogeny in the latter. Future simulation studies are thus recommended to better understand the impact of phylogenetic uncertainty and sampling design on diversification rate transitions.

The remarkable diversification of the Sigmodontinae involved significant paleogeographic changes at the continental and global scales. Mountain uplift and the progressive bridging of the Central American seaway, aided by sea level falls and a gradual cooling trend since the late middle Miocene, enabled the opportunistic invasion of South America by ancestral sigmodontines. The final closure of the Isthmus of Panama at ∼3.5 Ma (late Pliocene) [16], which triggered the onset of the main phase of the GABI [11] and possibly the reinvasion of Central and North America by some derived sigmodontine forms, is yet another episode in a series of events that marked the biogeographic history of the subfamily. Sigmodontine rodents successfully colonized the South American continent in the Neogene and in doing so they developed a wide array of ecomorphological specializations. Transitions in the tempo of diversification may explain the observed patterns of taxonomic richness, but further investigations are necessary to address the effect of shifting environmental conditions on species diversity and ecomorphology among different sigmodontine lineages.

## Supporting Information

Figure S1 **Time-calibrated tree topologies with nodal support for the phylogenetic relationships of sigmodontine rodents (plus other eumuroid taxa).** Node bars indicate 95% credibility intervals for the divergence dates of major clades listed in Table 1; (A) chronogram estimated in Multidivtime with bootstrap values; (B) chronogram estimated in BEAST with posterior probabilities.
(EPS)

Figure S2 **Divergence time estimates subjected to jackknife analyses of fossil calibrations.** Data points represent the variation of estimated dates using all set of 12 age constraints and when removing each fossil calibration numbered in Table S2.
(EPS)

Table S1 **Taxon sampling and classification scheme.** Muroid rodent taxa with GenBank accession numbers for the mitochondrial cytochrome *b* (*cytb*) and nuclear interphotoreceptor retinoid binding protein (*irbp*) genes used in this study. Sequence data obtained in this study (*).
(DOC)

Table S2 **Fossil records used as calibration points in molecular dating analyses.** Node numbers correspond to those depicted in Figure 1, with time units in million years.
(DOC)
