# Correction: In the Wake of Invasion: Tracing the Historical Biogeography of the South American Cricetid Radiation (Rodentia, Sigmodontinae)

**DOI:** 10.1371/journal.pone.0110081

**Published:** 2014-10-01

**Authors:** The PLOS ONE Staff

The following information is missing from the Funding section: CNPq grant (PDJ 150069/2014-6) to RNL.
