# Supplementary material for: In the Wake of Invasion: Tracing the Historical Biogeography of the South American Cricetid Radiation (Rodentia, Sigmodontinae)
Source: PLoS One. 2014 Jun 25;9(6):e100687. doi: 10.1371/journal.pone.0100687 (PMC4071052; doi:10.1371/journal.pone.0100687)
Supplement: Table S1 — Taxon sampling and classification scheme. Muroid rodent taxa with GenBank accession numbers for the mitochondrial cytochrome b (cytb) and nuclear interphotoreceptor retinoid binding protein (irbp) genes used in this study. Sequence data obtained in this study (*). (DOC) [file pone.0100687.s003.doc]

**Supporting Information table**

**Table S1. Taxon sampling and classification scheme.**

| **Taxon** | ***Cytb*** | ***Irbp*** |
| --- | --- | --- |
| Spalaciadae |  |  |
| *Myospalax aspalax* | AF326272 | AY326097 |
| Muridae |  |  |
| *Acomys spinosissimus* | AM409396 | AY326074 |
| Calomyscidae |  |  |
| *Calomyscus baluchi* | AY288509 | AY163581 |
| Nesomyidae |  |  |
| *Nesomys rufus* | AF160592 | AY326099 |
| Cricetidae |  |  |
| Arvicolinae |  |  |
| *Arvicola terrestris* | AY275106 | AY277407 |
| *Eothenomys melanogaster* | AM392374 | AY163583 |
| *Microtus oeconomus* | DQ452134 | AY163593 |
| *Myodes gapperi* | AY309431 | AY326080 |
| Cricetinae |  |  |
| *Cricetulus longicaudatus* | AJ973386 | AY326082 |
| *Cricetus cricetus* | AJ490302 | AY277410 |
| *Mesocricetus auratus* | AM904612 | AY163591 |
| *Phodopus sungorus* | AJ973390 | AY163631 |
| Neotominae |  |  |
| *Baiomys taylori* | AF548472 | AY277408 |
| *Neotoma albigula* | DQ179858 | AY277411 |
| *Neotoma lepida* | DQ179830 | AY163599 |
| *Onychomys torridus* | AY275110 | AY277412 |
| *Peromyscus maniculatus* | DQ385645 | AY163630 |
| *Peromyscus truei* | DQ861375 | AY277413 |
| *Reithrodontomys megalotis* | AF176248 | AY277414 |
| *Scotinomys teguina* | AF108705 | AY163639 |
| Tylomyinae |  |  |
| *Nyctomys sumichrasti* | AY195801 | AY163603 |
| *Tylomys nudicaudus* | DQ179812 | AY163643 |
| Sigmodontinae |  |  |
| Ichthyomyini |  |  |
| *Rheomys raptor* | KJ921706* | AY163635 |
| Sigmodontini |  |  |
| *Sigmodon alstoni* | AF293397 | AY163640 |
| *Sigmodon hispidus* | AF425227 | AY277479 |
| Oryzomyalia |  |  |
| Abrothrichini |  |  |
| *Abrothrix longipilis* | U03530 | AY163577 |
| *Abrothrix olivaceus* | AF297879 | AY277421 |
| *Chelemys macronyx* | U03533 | AY277441 |
| *Geoxus valdivianus* | AY275116 | AY277448 |
| *Notiomys edwardsii* | U03537 | AY163602 |
| *Pearsonomys annectens* | AF108672 | AY851749 |
| Akodontini |  |  |
| *Akodon azarae* | U03529 | AY163578 |
| *Akodon montensis* | AY273905 | AY277426 |
| *Bibimys labiosus* | DQ444329 | AY277436 |
| *Blarinomys breviceps* | AY275112 | AY277437 |
| *Brucepattersonius soricinus* | AY277486 | AY277439 |
| *Deltamys kempi* | AY195862 | AY277444 |
| *Juscelinomys huanchacae* | AY275119 | AY277452 |
| *Kunsia tomentosus* | AY275121 | AY277454 |
| *Lenoxus apicalis* | U03541 | AY277456 |
| *Oxymycterus nasutus* | AF175286 | AY277468 |
| *Necromys lasiurus* | AY273912 | AY277459 |
| *Necromys urichi* | AY273919 | AY277463 |
| *Scapteromys tumidus* | AY275133 | AY163637 |
| *Scapteromys aquaticus* | AY275132 | AY277476 |
| *Thalpomys cerradensis* | AY273916 | AY277480 |
| *Thaptomys nigrita* | AF108666 | AY277482 |
| Phyllotini |  |  |
| *Auliscomys pictus* | U03545 | AY277434 |
| *Calomys lepidus* | AF159294 | AY163580 |
| *Calomys callosus* | DQ447282 | AY277440 |
| *Eligmodontia typus* | AF108692 | AY277445 |
| *Graomys griseoflavus* | AY275117 | AY277449 |
| *Loxodontomys micropus* | AY275122 | AY277457 |
| *Phyllotis xanthopygus* | U86833 | AY163632 |
| Oryzomyini |  |  |
| *Cerradomys subflavus* | AF181274 | AY163626 |
| *Euryoryzomys macconnelli* | GU126538 | AY163620 |
| *Holochilus brasiliensis* | GU126517 | AY163585 |
| *Hylaeamys megacephalus* | AY275124 | AY163621 |
| *Melanomys caliginosus* | EU340020 | AY163590 |
| *Microryzomys minutus* | AF108698 | AY163592 |
| *Neacomys spinosus* | AF108701 | AY163597 |
| *Nectomys squamipes* | GU126522 | AY163598 |
| *Nephelomys albigularis* | GU126532 | AY163614 |
| *Nesoryzomys narboroughi* | GU126523 | AY163600 |
| *Nesoryzomys swarthi* | GU126524 | AY163601 |
| *Oecomys trinitatis* | GU126527 | AY163608 |
| *Oligoryzomys fulvescens* | GU126529 | AY163611 |
| *Oligoryzomys nigripes* | GU126530 | AY163612 |
| *Oreoryzomys balneator* | GU126535 | AY163617 |
| *Oryzomys couesi* | DQ185386 | AY163618 |
| *Oryzomys palustris* | GU126539 | AY163623 |
| *Pseudoryzomys simplex* | GU126547 | AY163633 |
| *Scolomys ucayalensis* | AF108696 | AY163638 |
| *Sooretamys angouya* | GU126534 | AY163616 |
| *Transandinomys talamancae* | GU126544 | AY163627 |
| *Zygodontomys brevicauda* | GU126549 | AY163645 |
| Reithrodontini |  |  |
| *Reithrodon auritus* | AY275129 | AY163634 |
| Thomasomyini |  |  |
| *Rhagomys longilingua* | AY206770 | DQ003723 |
| *Rhipidomys macconnelli* | AY275130 | AY277474 |
| *Rhipidomys nitela* | AF108682 | AY163636 |
| *Thomasomys aureus* | U03540 | AY277483 |
| *Thomasomys baeops* | DQ914654 | AY163642 |
| *Thomasomys ischyurus* | AF108675 | AY277484 |
| Wiedomyini |  |  |
| *Wiedomys pyrrhorhinos* | AY275134 | AY163644 |
| Incertae sedis |  |  |
| *Delomys sublineatus* | AF108687 | AY163582 |
| *Euneomys chinchilloides* | AY275115 | AY277446 |
| *Irenomys tarsalis* | U03534 | AY163587 |
| *Juliomys pictipes* | EF127514 | AY163588 |

Muroid rodent taxa with GenBank accession numbers for the mitochondrial cytochrome *b* (*cytb*) and nuclear interphotoreceptor retinoid binding protein (*irbp*) genes used in this study. Sequence data obtained in this study (*).
