# Supplementary material for: In the Wake of Invasion: Tracing the Historical Biogeography of the South American Cricetid Radiation (Rodentia, Sigmodontinae)
Source: PLoS One. 2014 Jun 25;9(6):e100687. doi: 10.1371/journal.pone.0100687 (PMC4071052; doi:10.1371/journal.pone.0100687)
Supplement: Table S2 — Fossil records used as calibration points in molecular dating analyses. Node numbers correspond to those depicted in Figure 1, with time units in million years. (DOC) [file pone.0100687.s004.doc]

**Supporting Information table**

**Table S**2. Fossil records used as calibration points in molecular dating analyses.

| **Node** | **Taxon** | **Fossil** | **Stage** | **Age** | **Reference** |
| --- | --- | --- | --- | --- | --- |
| 1 | Neotominae | *Paronychomys* spp. | Clarendonian | 11.6 |  |
| 2 | Cricetinae | *Mesocricetus primitivus* | MN 13 | 6.0 |  |
| 3 | Cricetinae | *Cricetus kormosi*, *Cricetus lophidens* | MN 13 | 6.0 |  |
| 4 | Arvicolinae | *Prosomys* (=*Promimomys*) *mimus* | early Ruscinian (MN 14); late Hemphillian | 5.5 |  |
| 5 | Sigmodontalia | *Prosigmodon oroscoi* | late Hemphillian | 4.8 |  |
| 6 | *Scapteromys* | *Scapteromys hershkovitzi* | Sanandresan | 2.2 |  |
| 7 | *Oxymycterus* | *Oxymycterus* cf. *rufus* | Ensenadan | 1.0 |  |
| 8 | Abrothrichini | ‘*Abrothrix*’ *kermacki* | late Chapadmalalan | 3.5 |  |
| 9 | Phyllotini | *Auliscomys* sp. | early Chapadmalalan | 4.0 |  |
| 10 | *Calomys* | *Calomys laucha* | Ensenadan | 1.0 |  |
| 11 | *Oligoryzomys* | *Oligoryzomys* cf. *flavescens* | late Ensenadan | 0.8 |  |
| 12 | *Holochilus* | *Holochilus* *brasiliensis*, *Holochilus* *primigenius* | late Ensenadan | 0.8 |  |

Node numbers correspond to those depicted in Figure 1, with time units in million years.

1. Baskin JA (1979) Small mammals of the Hemphillian age White Cone local fauna, northeastern Arizona. Journal of Paleontology 53: 695-708.

2. Carleton MD, Musser GG (1984) Muroid rodents. In: Anderson S, Jones Jr. JK, editors. Orders and Families of Recent Mammals of the World. New York: John Wiley & Sons Inc.

3. Jacobs LL (1977) Rodents of the Hemphillian age Redington local fauna, San Pedro Valley, Arizona. Journal of Paleontology 51: 505-519.

4. Jacobs LL, Lindsay EH (1984) Holoartic radiation of Neogene muroid rodents and the origin of South American cricetids. Journal of Vertebrate Paleontology 55: 265-272.

5. Korth WW (1994) The Tertiary Record of Rodents in North America; Stehli FG, Jones DS, editors. New York: Plenum Press.

6. McKenna MC, Bell SK (1997) Classification of Mammals above the Species Level. New York, NY: Columbia University Press.

7. Savage DE, Russell DE (1983) Mammalian Paleofaunas of the World. Reading: Addison-Wesley Publishing Co.

8. de Bruijn H, Dawson MR, Mein P (1970) Upper Pliocene Rodentia, Lagomorpha and Insectivora (Mammalia) from the Isle of Rhodes (Greece). I, II and III. Proceedings of the Koninklijke Nederlandse Akademie van Wetenschappen, B 73: 535-584.

9. Freudenthal M, Mein P, Martín-Suárez E (1998) Revision of late Miocene and Pliocene Cricetinae (Rodentia, Mammalia) from Spain and France. Treballs del Museu de Geologia de Barcelona 7: 11-93.

10. Agustí J, Cabrera L, Garcés M, Krijgsman W, Oms O, et al. (2001) A calibrated mammal scale for the Neogene of Western Europe. State of the art. Earth-Science Reviews 52: 247-260.

11. Chaline J, Brunet-Lecomte P, Montuire S, Viriot L, Courant F (1999) Anatomy of the arvicoline radiation (Rodentia): palaeogeographical, palaeoecological history and evolutionary data. Annales Zoologici Fennici 36: 239-267.

12. Martin RA, Goodwin HT, Farlow JO (2002) Late Neogene (late Hemphillian) rodents from the Pipe Creek Sinkhole, Grant County, Indiana. Journal of Vertebrate Paleontology 22: 137-151.

13. Repenning CA (2003) Chapter 17: *Mimomys* in North America. Bulletin of the American Museum of Natural History 279: 469-512.

14. Jacobs LL, Lindsay EH (1981) *Prosigmodon oroscoi*, a new sigmodont rodent from the late Tertiary of Mexico. Journal of Paleontology 55: 425-430.

15. Peláez-Campomanes P, Martin RA (2005) The Pliocene and Pleistocene history of cotton rats in the Meade basin of southwestern Kansas. Journal of Mammalogy 86: 475-494.

16. Quintana CA (2002) Roedores cricétidos del Sanandresense (Plioceno tardío) de la provincia de Buenos Aires, Argentina. Mastozoología Neotropical 9: 263-275.

17. Pardiñas UFJ, D'Elía G, Ortiz PE (2002) Sigmodontinos fósiles (Rodentia, Muroidea, Sigmodontinae) de América del Sur: estado actual de su conocimiento y prospectiva. Mastozoología Neotropical 9: 209-252.

18. Pardiñas UFJ, Tonni EP (1998) Procedencia estratigráfica y edad de los más antiguos muroideos (Mammalia, Rodentia) de América del Sur. Ameghiniana 35: 473-475.

19. Reig OA (1978) Roedores cricétidos del Plioceno superior de la provincia de Buenos Aires (Argentina). Publicación del Museo Municipal de Ciencias Naturales Lorenzo Scaglia 2: 164-190.

20. Voglino D, Pardiñas U (2005) Roedores sigmodontinos (Mammalia: Rodentia: Cricetidae) y otros micromamíferos pleistocénicos del norte de la provincia de Buenos Aires (Argentina): reconstrucción paleoambiental para el Ensenadense cuspidal. Ameghiniana 42: 143-158.

21. Pardiñas UF, Teta P, Voglino D, Fernandez FJ (2013) Enlarging rodent diversity in west-central Argentina: a new species of the genus *Holochilus* (Cricetidae, Sigmodontinae). Journal of Mammalogy 94: 231-240.
